# Antimicrobial resistance in livestock: antimicrobial peptides provide a new solution for a growing challenge

**DOI:** 10.1093/af/vfy005

**Published:** 2018-05-08

**Authors:** Zhi Li, Yuhan Hu, Yuanyuan Yang, Zeqing Lu, Yizhen Wang

**Affiliations:** Laboratory of Animal Nutrition and Feed Science, Department of Animal Science, Zhejiang University, Hangzhou, Zhejiang Province, China

**Keywords:** antibiotic alternatives, antimicrobial peptides, antimicrobial resistance, livestock

**Implications**

- The rise in antimicrobial resistance is widely acknowledged as a result of misuse and overuse of livestock antibiotics. Once transferred to human beings, these strains can cause diseases that are not treatable by antibiotics.
- Antibiotics can largely accumulate in the environment through livestock excretion and eventually threaten public health.
- Current government regulations on antibiotics cannot effectively and immediately stop the increase in antimicrobial resistance. There is an urgent need to find alternatives that do not lead to resistance in the future.
- Antimicrobial peptides hold promise as effective alternative as they have efficacious antimicrobial effects and weak resistance induction ability.

## Introduction

Animal husbandry, the agricultural practice of breeding and raising livestock, is a major food-producing industry worldwide. Compromised gut health due to improper nutrition and management is a significant challenge for this industry as it can result in poor development and growth, disease, morbidity, and mortality in livestock. Poor gut health is often associated with leaky gut, intestinal atrophy, infection, and inflammation, particularly when animals are young. Antibiotics are widely used in modern livestock production as growth promoters primarily due to their preventative effects on livestock diseases. Global consumption of antibiotics from food animal production was estimated to be 63,151 tons in 2010 and is predicted to rise dramatically by 67% by 2030 (Van Boeckel et al., 2015). This practice has been linked to the spread of antimicrobial-resistant pathogens in both livestock and humans, posing a significant public health threat. Antimicrobial resistance is becoming a worldwide concern, and without effective countermeasures, it is predicted to kill more than 10,000,000 human beings and cause $100 trillion economic loss annually by 2050 (O’Neil, 2014). Due to the widespread use of antibiotics for treatment, prophylaxis, and growth promotion, livestock have become a reservoir of antimicrobial-resistant bacterial strains and genes. According to prevalence studies and homology sequence analyses, resistant strains have been extensively identified in animals, and they can be transferred to farmers, slaughterhouse workers, and veterinarians through direct contact, and even consumers through the food chain (Marshall and Levy, 2011; Vishnuraj et al., 2016). Concerns about this issue have risen rapidly, with a surge of published articles in the past 10 yrs (from 4 to 73, based on a search of “antimicrobial resistance” and livestock in PubMed database). Notably, resistance to the last-resort antibiotic, colistin, has been found in livestock, alarming us to the impending threat of antimicrobial resistance (Irrgang et al., 2016; Liu et al., 2016). Moreover, antibiotic residues in excrement may cause a high level of antibiotic accumulation in the environment, increasing the exposure and occurrence of antibiotic resistance, and leading to more profound and complex impacts.

In the face of the antimicrobial resistance threat, authorities of major economies, including the European Medicines Agency and the Food and Drug Administration (FDA) in the United States, have imposed regulations on antibiotic growth promoters (**AGP**) and have tried to put treatment antibiotics under official surveillance to decrease antimicrobial resistance through reduced antibiotic use. However, withdrawal of antibiotics from livestock productions can result in a number of challenges, including compromised gut health and a rise in gut diseases. Concurrently a number of effective alternatives have also been reported; for example, antimicrobial peptides, which play multiple roles in bacterial elimination, immune response, epithelial reinforcement, and combating diseases like cancer (Chu et al., 2015; de la Fuente-Núñez et al., 2017). Antimicrobial peptides further show biofilm destruction on multi-resistant strains while rarely resulting in antimicrobial resistance (Ageitos et al., 2017). Antimicrobial peptides can exhibit favorable therapeutic effects on piglet diarrhea depending on direct antimicrobial effects, intestinal barrier enhancement, and inhibition of inflammation (Yi et al., 2016, 2017). In this review, we summarize the current status of antibiotic use in livestock production and potential challenges associated with the use of antibiotics and then elaborate on the antimicrobial effects of antimicrobial peptides, their underlying mechanisms, and the potential of antimicrobial peptides in livestock production.

## Threats from Antibiotics Use in Livestock Production

### Selection of antimicrobial-resistant bacteria

The livestock industry is observing rising levels of antimicrobial resistance, similar to what has been happening to antimicrobial resistance findings in human clinical isolates (O’Neill, 2015a). Based on EU official statistics, we made a summary about antimicrobial resistance dynamics of the most prevailing food-borne pathogens: *Escherichia coli* and *Salmonella* spp. isolated from pigs, cattle, and broilers (Tables 1 and 2). Antibiotics ranked as critically important or important to human medicine by WHO are ones that become less effective through inappropriate use and in turn lead to failures of bacterial disease treatment. Through both rigid restrictions and public awareness, the goal is to see a decrease, or at least delay, in antimicrobial resistance development. However, the current moment is critical, as evidenced by *E. coli* data that shows an obvious upward trend in antimicrobial resistance based on a high level in pigs and broilers, while resistant ratio in *Salmonella* is much smaller and the rising trend in resistance to some antibiotics is partially reverted. Remarkably, resistance to our last-resort antibiotic, colistin, is commonly detected in both bacteria from the tested species. Considering strict regulations on the use of antibiotics in livestock production in the EU, the survey indicates that current policies may not be effective enough to tackle increased antimicrobial resistance.

**Table 1.** *Escherichia coli* antimicrobial resistance (%) isolated from food-producing animals in European Union

Category	Year	AMP	CTX	CHL	CIP	CST	GEN	NAL	SXT	TET
Pig	2010	21.0	1.0	7.0	2.0	-	2.0	2.0	37.0	48.0
	2015	39.3	1.4	18.3	10.5	0.4	3.3	6.0	44.2	54.7
Cattle	2010	28.0	3.0	17.0	15.0	-	9.0	13.0	34.0	38.0
	2015	31.0	1.7	15.4	11.4	0.9	3.8	8.7	36.6	45.4
Broiler	2010	35.0	5.0	8.0	29.0	-	4.0	26.0	34.0	31.0
	2014	58.6	5.1	21.6	65.7	0.9	11.6	62.6	53.1	50.1

Data from The European Union Summary Report on antimicrobial resistance in zoonotic and indicator bacteria from humans, animals, and food in 2010, 2014, and 2015.

AMP, ampicillin; CTX, cefotaxime; CHL, chloramphenicol; CIP, ciprofloxacin; CST, colistin; GEN, gentamicin; NAL, nalidixic acid; SXT, sulfamethoxazole; TET, tetracycline.

**Table 2.** *Salmonella* antimicrobial resistance (%) isolated from food-producing animals in European Union

Category	Year	AMP	CTX	CHL	CIP	CST	GEN	NAL	SXT	TET
Pig	2010	21.0	1.0	7.0	2.0	-	2.0	2.0	37.0	48.0
	2015	39.3	1.4	18.3	10.5	0.4	3.3	6.0	44.2	54.7
Cattle	2010	28.0	3.0	17.0	15.0	-	9.0	13.0	34.0	38.0
	2015	31.0	1.7	15.4	11.4	0.9	3.8	8.7	36.6	45.4
Broiler	2010	35.0	5.0	8.0	29.0	-	4.0	26.0	34.0	31.0
	2014	58.6	5.1	21.6	65.7	0.9	11.6	62.6	53.1	50.1

Data from The European Union Summary Report on antimicrobial resistance in zoonotic and indicator bacteria from humans, animals, and food in 2010, 2014, and 2015.

AMP, ampicillin; CTX, cefotaxime; CHL, chloramphenicol; CIP, ciprofloxacin; CST, colistin; GEN, gentamicin; NAL, nalidixic acid; SXT, sulfamethoxazole; TET, tetracycline.

### Antibiotic residue in the environment

Antibiotics are not easily degraded in the animal body and thus enter into the environment through excreted urine and feces (Lim et al., 2013). Due to the farming scale, livestock farms have been reported to discharge a large amount of antibiotics into nearby surface water, soil, and sludge (Watanabe et al., 2010, Zhu et al., 2013). In China, 84.0% of total antibiotic excretion comes from farming animals (pig: 44.4%, chicken: 18.8%, other animals: 20.9%; Zhang et al., 2015). Residual antibiotics usually have negative impacts on organisms, food security, and water security. These can result in a selection of antimicrobial strains and accumulation in the human body through the food chain and drinking water (Sarmah et al., 2006; Näslund et al., 2008; Underwood et al., 2011; Qian et al., 2012; Boonsaner and Hawker, 2013; Jia et al., 2017). According to a comprehensive survey of total antibiotic emissions in river basins of China, an overlap with animal industry area and the basin province is clearly evident: the areas with a high level of antibiotic residue produce nearly 1/2 of all slaughter hogs and 1/3 of overall meat (Zhang et al., 2015). Antibiotic residues from livestock also occur in some developed countries. Oxytetracycline and sulfadiazine are antibiotics commonly used in livestock and can be detected to quantify the animal contribution. Kolpin et al. (2002) carried out a survey of 139 streams in the United States, and the maximum concentration of oxytetracycline was 340 ng/L. In the United Kingdom, the maximum concentrations of the two antibiotics were 4490 and 4130 ng/L, respectively (Environmental Agency, 2005). Although there is a lack of recent survey data about antibiotic residue in water, we speculate that there should be a rising trend in the natural environment in recent years as a result of the worldwide boost in animal product consumption, the corresponding growth in farming size, and our current sewage treatment methods.

## Solutions to the Threats Caused by Antibiotic Use in Livestock Production

### Official regulation and surveillance

**Figure 1.**
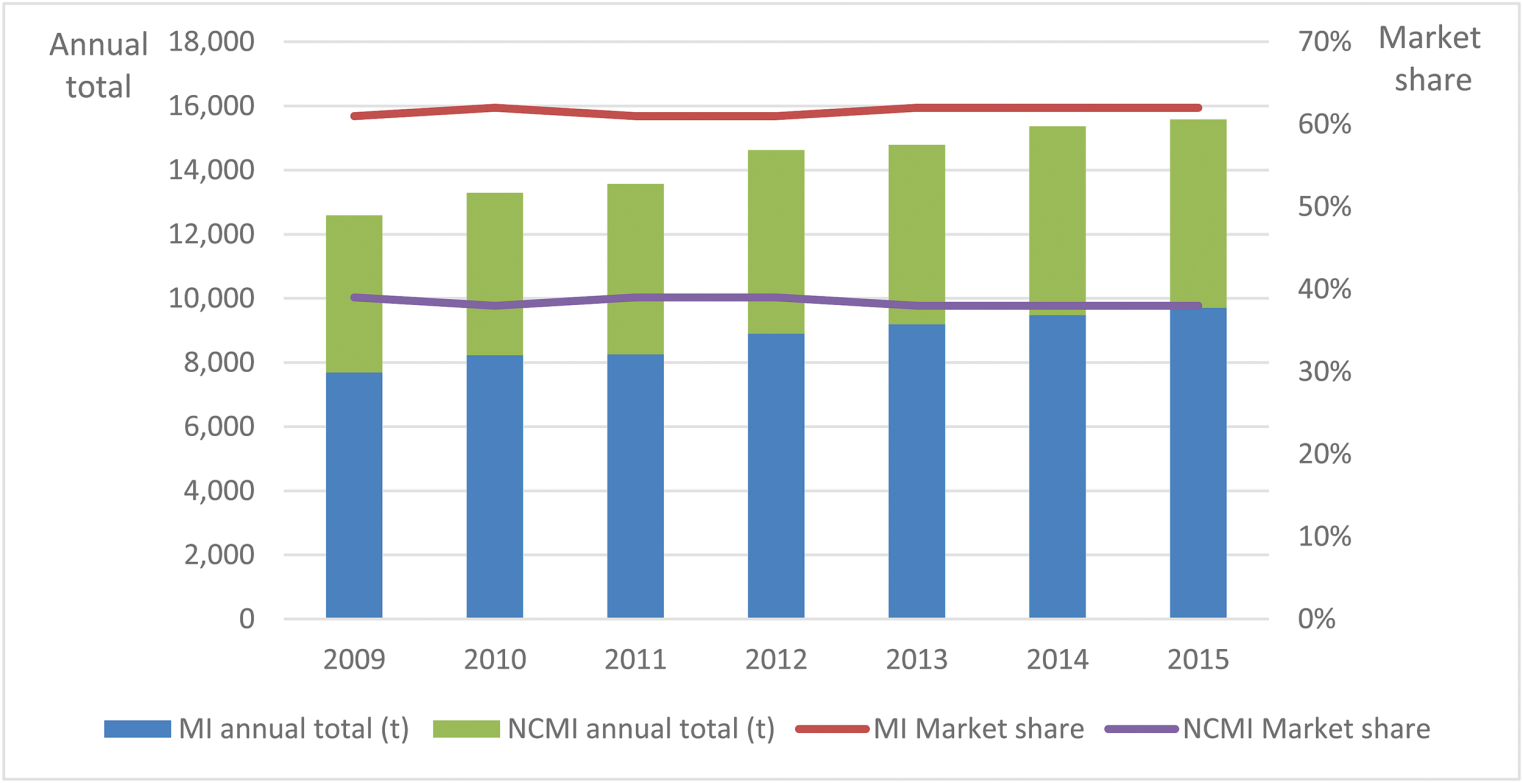
Antimicrobials sold in the United States classified by medical importance. MI, medically important; NCMI, not currently medically important. Data from antimicrobials sold or distributed for use in food-producing animals from (issued by FDA of the United States) 2010 to 2015.

Facing the serious threat of livestock-use antimicrobial drugs, the governments of major economies have already imposed a series of policies, acts, and guidelines to ensure the responsible use of antibiotic drugs. In the United States, the FDA issued a series of documents concerning antimicrobial use in food-producing animals, claiming that until 2016 no antibiotics deemed medically important can be used as AGP. However, annual sales of antibiotics from 2009 to 2015 grew dramatically, with little changes in market ratios of medically important and not currently medically important antibiotics (Figure 1).

As one of the largest pork exporters, Denmark has taken serious steps in tackling antimicrobial resistance by becoming one of the first countries to ban all AGP. AGP were gradually banned from 1995 to 2000 (Jensen and Hayes, 2014) after the Denmark government introduced a Yellow Card scheme to punish farmers using excessive prescriptions. The antibiotic restrictions proved to be successful with regard to antimicrobial consumption and economic income (O’Neill, 2015b). As for the primary purpose of imposing antimicrobial resistance control, however, it is a little disappointing to stop the uprising trend of antimicrobial resistance through regulations on consumption and using methods (Jensen and Hayes, 2014). Together with increased morbidity in piglets, the ban on AGP and antibiotic limitations requires better management and feeding practices, even in Denmark, a developed country with rich experience and technical talent (Jensen and Hayes, 2014).

In spite of the governmental progress aimed at controlling livestock-use antibiotics, there is a lack of a unified classification for antibiotics, which continues to exacerbate issues of overuse and misuse. The U.S. FDA has ranked the importance of antibiotics into three categories: critically important, highly important, and important. The WHO, however, only has two classification levels: critically important and highly important. There are also some differences in the detailed items. In addition, no unified evaluation system is widely executed for antimicrobial resistance regulation. These multiple and somewhat incongruous criteria also complicate antimicrobial resistance surveillance and antibiotic drug regulation.

### Alternatives to antibiotics

The current solutions to antimicrobial resistance for livestock-use antibiotics, such as a ban on AGP and strict regulations on veterinary use, proved to be unsatisfactory when evaluated based on antimicrobial resistance change. To avoid the negative effects of antibiotics, novel alternative approaches to antibiotics should not easily induce resistance in bacteria. Additionally, the absolute replacement of antibiotics is not realistic in the near future. Preventive therapies or adjunctive treatments with antibiotics that enhance the efficacy and consequently reduce normal doses should be regarded as an ideal approach. According to this standard, there are nine categories (antibodies, probiotics, lysins, wild-type or engineered bacteriophages, immune enhancers, vaccines, antimicrobial peptides, host defense peptides, and antibiofilm peptides) that have great promise for commercialization in the next decade (Czaplewski et al., 2016). Except for lysins, bacteriophages, host defense peptides, and antibiofilm peptides, each alternative has two development directions. Besides, some alternative categories are partially overlapped, for example, immune stimulation, host defense peptides, innate defense peptides, and antibiofilm peptides usually function through antimicrobial peptides. Due to public awareness of antibiotic-related issues and according to business requirements, this list is rapidly expanding through the continuous efforts of academic institutions and pharmaceutical corporations. Antibiotic alternative research is a hot topic and requires further development in the pharmaceutical pipeline. Recently one product, Bezlotoxumab, was approved by the U.S. FDA in 2016 as an anti-infection drug.

## Antimicrobial Effects of Antimicrobial Peptides and Their Modes of Action

### Antimicrobial effects of antimicrobial peptides

Antimicrobial peptides, also known as antibacterial peptides or host defense peptides, are a 5,000-member family of short cationic peptides (less than 100 amino acids) which constitute part of the innate immune defense existing in nearly all classes of organisms (Ganz, 2002; Waghu et al., 2014; Ageitos et al., 2017). All of them commonly share a small size, linear or cyclic structure, and enjoy an 80-yr application history (Dubos and Cattaneo, 1939; Boman, 1995).

Of all the bioactivities discovered, antimicrobial effect is the first discovered and consequently drew the most attention with regard to antibiotic alternatives (STEINER et al., 1981). In terms of minimal inhibitory concentration, antimicrobial peptides exhibit significantly high effective inhibition on bacteria (Table 3). Although smaller minimal inhibitory concentrations do not necessarily mean reduced antimicrobial resistance occurrence, it can at least decrease the likelihood of unnecessary contact between targeted bacteria and antimicrobials. Secondly, antimicrobial peptides usually have a broad-spectrum activity against either Gram＋ or Gram－ bacteria. Among 136 natural antimicrobial peptides from microorganisms, aquatic organisms, and terrestrial organisms, 90 antimicrobial peptides (66.2%) have inhibitory effects on both Gram＋ and Gram－, and 23 (16.9%) and 23 (16.9%) antimicrobial peptides show single effects on Gram＋ and Gram－ bacteria, respectively (Ageitos et al., 2017).

**Table 3.** Minimal inhibitory concentration of selected antimicrobial peptides toward food-borne pathogens

Antimicrobial peptides	Minimal inhibitory concentration (μM)				References
	*Escherichia coli*	*Salmonella*	*Staphylococcus aureus*	*Bacillus subtilis*	
Gageotetrins	-	0.02 to 0.06			Tareq et al. (2014)
Discodermin A	1	-	1	2	Matsunaga et al. (1984)
Thanatin	<1.2	<1.2	-	<5	Fehlbaum et al. (1996)
Maximin 3	0.3	-	1.1	-	Lai et al. (2002)
CPF-St5	1	-	1	-	Roelants et al. (2013)
buCATHL4C	12.5	-	0.2 to 0.4	-	Brahma et al. (2015)
Indolicidin	0.8	-	3	-	Falla et al. (1996)
TAP	3 to 6	-	6 to 12	-	Diamond et al. (1991)
GNCP-1	5	-	3	-	Yamashita and Saito (1989)
HBD-3	1	-	0.6	-	Joly et al. (2004)
HNP-1	0.5	-	0.6	1.9	De Smet and Contreras (2005)
LL-37	0.1	0.4	-	-	Turner et al. (1998); De Smet and Contreras (2005)

Furthermore, antimicrobial peptides can enhance the efficacy of antibiotics while reducing their concentration, particularly on antimicrobial-resistant strains. Soren et al. investigated the combinative effects of novicidin with rifampin, ceftriaxone, or ceftazidime, on corresponding antibiotic-resistant bacterial strains. All formulas show synergistic effects on resistant bacteria with 70%, 89.7%, and 94.1% isolates inhibited, respectively, with reduced MIC and less hemolytic activity, whose synergetic effect is related to cytoplasmic membrane damage induced by novicidin (Soren et al., 2015). Similar results were found in combinations of azithromycin and LL-37 or colistin on multi-drug-resistant isolates (Lin et al., 2015).

### Mechanisms for the antimicrobial properties of antimicrobial peptides

Mechanisms for the antimicrobial properties of antimicrobial peptides are like a pipeline composed of initial attraction and interaction, concentration-dependent threshold, self-association and multimerization, antimicrobial actions, followed by induced cellular activities (Lee et al., 2016). Inasmuch, antimicrobial models are the most extensively investigated, with 21 models used to elucidate antimicrobial peptides’ damage on bacteria. These models can be divided into a non-permeabilizing category (2 models) and a permeabilizing category (19 models), the latter of which can be further classified by pore formation or not (Wang et al., 2015). For example, two main models, barrel-stave and toroidal wormhole, are attributed to pore formation. In the barrel-stave model, ampicillin (AMP) will transform and acquire amphipathy after binding to a membrane. During accumulation to threshold concentration, antimicrobial peptides will gradually form into bigger molecules by which peptides can inset more deeply and form a “barrel,” a ring-like pore, and a “stave,” which is made of individual spokes around the ring. In this model, the hydrophobic domains locate outward and the hydrophilic regions locate inward, avoiding exposure to residues with the opposite hydrophilic preference (Breukink and de Kruijff, 1999). The toroidal wormhole model is similar to the barrel-stave model and is mainly reported in helical antimicrobial peptides (Matsuzaki, 1998; Hara et al., 2001; Hara et al., 2001). However, in this model, AMP hydrophobic residues are not exposed to the lipid head groups in the cell membrane (Zhu et al., 2015). Carpet mechanism does not rely on pore formation and will non-specifically cover the surface like a carpet (Taubes, 2008), whereas it is sometimes regarded as a final step in the toroidal wormhole model (Fernandez et al., 2012). Along with membrane damage, many important pathways are unable to function (Melo et al., 2009). All of these steps take place in a few minutes, while it takes days for antibiotics to exert their antimicrobial effects (Tossi et al., 1997).

## Prospective of Antimicrobial Peptides as Antimicrobial Drugs

### Improvement and production

Further therapeutic development still requires all-round improvements in antimicrobial efficiency, cytotoxicity, and stability in physiological conditions. Due to the biofilm destruction, which is a lack of distinguishable markers, the antimicrobial effect of natural antimicrobial peptides usually combines with an attack on host cells (Lee et al., 2016). In addition, the greatly decreased bioactivity in plasma is another issue that obstructs the medical practice. LL-37, for example, exerts less than 1/64 bioactivity toward *Staphylococcus aureus* in the plasma-simulated solution (plasma:PBS = 1:1; de Breij et al., 2018). Improvements on the mentioned sides are usually via: 1) hybrids of active fragments from different antimicrobial peptides, 2) antimicrobial peptides modifications, 3) innovations not based on natural antimicrobial peptides, and 4) computer-aided technology based on an antimicrobial peptides database.

The hybrid method is mostly applied in antimicrobial peptide synthesis aimed at increasing antimicrobial efficacy, changing targeted bacteria, and compromising cytotoxicity (Wade et al., 1990). CAMEL0 is a hybrid of cecropin A and melittin A with lower minimal inhibitory concentration level (*E. coli*, 1 μM) compared with original melittin (Gram＋/Gram－, 1 to 8 μM; Oh et al., 2000; Park et al., 2011). RW-BP100, a modified protein based on cecropin A-melittin hybrid protein, has enhanced effects on both Gram＋ (minimal inhibitory concentration 0.3 to 1 μM) and Gram－ bacteria (minimal inhibitory concentration 0.1 to 2.5 μM), but with greater cytotoxicity (Badosa et al., 2007; Torcato et al., 2013). RN7-IN6 is an improved hybrid of indolicidin and ranalexin with more potent efficacy than indolicidin and ranalexin.

Antimicrobial peptide modification happens on residues of only one kind of AMP. Pexiganan is an analog of magainin 2, showing a wide antimicrobial spectrum. A total number of 3,108 bacteria strains can be inhibited, among which 87% minimal inhibitory concentration ranges from 0.8 to 6.5 μM. More importantly, the strains are mostly resistant to antibiotics of medical importance to human beings (Gottler and Ramamoorthy, 2009). Other modified antimicrobial peptides, like Dhvars (originated from histatin; Welling et al., 2007), FL9 (originated from Fallaxin; Gottschalk et al., 2015), and WMR-NH2 (originated from Myximidin; Cantisani et al., 2014), show outstanding inhibitory effects, particularly on drug-resistant strains.

As for the innovated antimicrobial peptides, they have more precise targets and surprising inhibitory effects. V-peptide, targeted on lipopolysaccharide and the constituent lipid A, is able to inhibit Gram－ strains at minimal inhibitory concentration between 0.004 and 0.692 μM (Frecer et al., 2004). WLBU2 can inhibit bacteria at a low minimal inhibitory concentration regardless of the salt concentration. Applications of computer-aided designs represent the next generation highlighted by high potency and precise prediction based on enriching AMP databases like APD3, CAMPR3, and LAMP (Zhao et al., 2013; Waghu et al., 2016; Wang et al., 2016).

Currently, antimicrobial peptides are generally produced by chemical methods, not by bioreactors, due to the cytotoxicity produced in the latter pipeline. Even so, some antimicrobial peptides, including LL-37 and β-defensins, have been successfully expressed in *E. coli*. As a promising antibiotic alternative, in both humans and animals, it requires further breakthroughs in production method, microbial efficacy, security, and acceptable cost.

### Delivery methods

As an important step in pharmaceutical research, it is necessary to investigate the delivery systems that carry antimicrobial peptides to targeted sites. However, very few articles focus on this topic, which is unfortunate given its role in enhancing antibacterial effects, reducing degradation, controlling release rate, and decreasing cytotoxicity.

Up to now, inorganic and organic materials are the two main kinds of delivery method. Inorganic delivery materials include mesoporous silica, titanium, metal nanoparticles, quantum dots, carbon-based nanoparticles, and related materials. Mesoporous silica shows good incorporation with antimicrobial peptides like LL-37, which enhances stability against degradation, specific adsorption, extended release period, and synergetic effects with antimicrobial peptides (Braun et al., 2016). The synergetic effects are probably related to surface enrichment in the membrane resulting in membrane disruption, and therefore, minimal inhibitory concentration will be lower than normal (Li and Wang, 2013). Apart from most of the mentioned characteristics, titanium materials can markedly reduce the cytotoxicity of some antimicrobial peptides (Kazemzadeh-Narbat et al., 2013). Metal nanoparticles researched are mainly Au, Pt, Ag, and Cu and have highlighted enhancement of antimicrobial effects. For example, when delivered by gold nanodots, surfactin minimal inhibitory concentration toward methicillin-resistant *S. aureus* can be lowered by more than 80-fold (Nordström and Malmsten, 2017). This may be a result of released ions and induction of reactive oxygen species (Hajipour et al., 2012). Quantum dot is reported to be system-specific, varying in cytotoxicity and antimicrobial effects (Park et al., 2011; Chen et al., 2015; Galdiero et al., 2016). Carbon-based nanomaterials, including graphene and carbon tubes, are drawing more attention because of their imposed membrane damage and induction of oxidative stress (Hajipour et al., 2012), which can also enhance antimicrobial activity (Nellore et al., 2015).

Organic delivery materials, or polymeric materials, include particles and fibers, gels, multilayers, and conjugates. Beyond their own antimicrobial and synergetic effects, organic materials also possess outstanding plasticity and can be designed according to specific requirements. For example, polymer multilayers can be used to build more complex particles and surface coatings. Since materials in solution are gradually removed in the deposition process, it can sustain ambient conditions and the various parameters can be varied between each layer (Nordström and Malmsten, 2017).

### Endogenous AMP regulation

Due to its excellent antimicrobial effects and other activities in immune enhancement, endogenous AMP regulation through nutrients or other chemicals is developed as a prevention method. Considering its oral administration method and high security (nutrients in feed), it would be convenient to use in a whole herd as prophylaxis. Butyrate and its derivatives, including 4-phenylbutyrate and sodium butyrate, show improvements in endogenous AMP LL-37 expression in both epithelial cells and macrophages (Schauber et al., 2003; Raqib et al., 2006; Steinmann et al., 2009a). Vitamin D can trigger expression of LL-37 in epithelial tissue as well as leukocytes, which was applied in treatment to *Mycobacterium tuberculosis* infections (Zasloff, 2006). A derivative of Vitamin D, 1, 25-dihydroxyvitamin D3, has similar enhanced effects on LL-37 and exhibits synergetic effects with 4-phenylenediamine (Gombart et al., 2005; Steinmann et al., 2009b). Stimulator aroylated phenylenediamine is the latest discovery and has shown significant stimulation in LL-37 synthesis by 20- to 30-fold (Ottosson et al., 2016). Despite its comparatively short history, it has already been listed as one of the most promising antibiotic alternatives (Czaplewski et al., 2016).

## Conclusion

Antimicrobial resistance poses a worldwide threat to public health, which may be partially associated with using AGP in livestock production. The European Union banned the use of AGP in animal food production in 2006. The U.S. FDA placed restrictions on antibiotic use in animal production in December 2016; more countries are expected to follow. However, a withdrawal of antibiotics from livestock production can result in a number of challenges, including a rise in gut diseases. Therefore, the development of antibiotic alternatives for sustainable livestock production is urgently needed as the livestock industry complies with these new regulations. Antimicrobial peptides represent one of the most promising alternatives to antibiotics, with several beneficial properties including a lower risk of inducing antimicrobial resistance, excellent inhibitory effects, easiness of degradation, and host immunity enhancement. However, their application in livestock production has been limited, largely due to some evidenced disadvantages, including stability, susceptibility to proteolysis, low activity under physiological conditions, and a high cost of production. Their inconsistent efficacy and the only partial understanding of their modes of action have also prevented antimicrobial peptides from reaching the market place. A better understanding of the effects of antimicrobial peptides on the three components of the gastrointestinal ecosystem—gut microbiota, gut physiology, and immunology—and the mechanisms behind them will possibly allow us to make the better use of antimicrobial peptides for economically effective and sustainable livestock production. Proper delivery methods, including microencapsulation and nanotechnology, provide promising tools to deliver antimicrobial peptides to the animal gut and improve the efficacy of antimicrobial peptides in livestock production. Finally, the potential risks in using antimicrobial peptides for livestock production need to be evaluated systemically.

## Funding

This work was supported by a grant from the Key Program of National Natural Science Foundation of China (grant no. 3163000269), Modern Agricultural Industry Technology System (grant no. CARS-35), Major Science and Technology Projects of Zhejiang Province (grant no. 2015C02022).

## About the Authors

**Dr Yizhen Wang** has been awarded with Qiushi Professor at Zhejiang University. During the 30-year career, he devoted in biological feed stuffs, animal nutrition, immune regulation, and meat quality improvement. He has published more than 110 SCI articles including *Nature Communications*, *Biomaterials*, *Journal of Immunology* and *Journal of Animal Science*. In practice, Wang has been authorized with 17 patents, produced 3 national criteria, and developed 7 feed additive products.

**Zeqing Lu** was awarded his Ph.D. from Zhejiang University and is now a mater supervisor at the Institute of Feed Science, Zhejiang University. He specializes in alternative and deducted methods of livestock-use antibiotics and reduction of heavy metal supplementation.

**Zhi Li** is a Ph.D. candidate under the supervision of Professor Yizhen Wang in Institute of Feed Science, Zhejiang University.

**Yuhan Hu** is currently studying for a Master’s Degree at the Institute of Feed Science, Zhejiang Univer sity. She majors in microbial polysaccharides under the supervision of research assistant Zeqing Lu.

## References

[CIT0001] Ageitos J.M., Sánchez-Pérez A., Calo-Mata P., and Villa T.G. 2017 Antimicrobial peptides (amps): ancient compounds that represent novel weapons in the fight against bacteria. Biochem. Pharmacol. 133:117–138. doi:10.1016/j.bcp.2016.09.018. 27663838 10.1016/j.bcp.2016.09.018

[CIT0003] Badosa E., Ferre R., Planas M., Feliu L., Besalú E., Cabrefiga J., Bardají E., and Montesinos E. 2007 a library of linear undecapeptides with bactericidal activity against phytopathogenic bacteria. Peptides 28:2276–2285. doi:10.1016/j.peptides.2007.09.010. 17980935 10.1016/j.peptides.2007.09.010

[CIT0004] Boman H.G 1995 Peptide antibiotics and their role in innate immunity. Annu. Rev. Immunol. 13:61–92. doi:10.1146/annurev.iy.13.040195.000425. 7612236 10.1146/annurev.iy.13.040195.000425

[CIT0005] Boonsaner M., and Hawker D.W. 2013 Evaluation of food chain transfer of the antibiotic oxytetracycline and human risk assessment. Chemosphere 93:1009–1014. doi:10.1016/j.chemosphere.2013.05.070. 23790827 10.1016/j.chemosphere.2013.05.070

[CIT0006] Brahma B., Patra M.C., Karri S., Chopra M., Mishra P., De B.C., Kumar S., Mahanty S., Thakur K., Poluri K.M., et al 2015 Diversity, antimicrobial action and structure-activity relationship of buffalo cathelicidins. PLoS One 10:e0144741. doi:10.1371/journal.pone.0144741. 26675301 10.1371/journal.pone.0144741 PMC4684500

[CIT0007] Braun K., Pochert A., Lindén M., Davoudi M., Schmidtchen A., Nordström R., and Malmsten M. 2016 Membrane interactions of mesoporous silica nanoparticles as carriers of antimicrobial peptides. J. Colloid Interface Sci. 475:161–170. doi:10.1016/j.jcis.2016.05.002. 27174622 10.1016/j.jcis.2016.05.002

[CIT0008] de Breij A., Riool M., Cordfunke R.A., Malanovic N., de Boer L., Koning R.I., Ravensbergen E., Franken M., van der Heijde T., Boekema B.K., et al 2018 The antimicrobial peptide SAAP-148 combats drug-resistant bacteria and biofilms. Sci. Transl. Med. 10. 10.1126/scitranslmed.aan4044 29321257

[CIT0009] Breukink E., and de Kruijff B. 1999 The lantibiotic nisin, a special case or not?Biochim. Biophys. Acta 1462:223–234. 10590310 10.1016/s0005-2736(99)00208-4

[CIT0010] Cantisani M., Finamore E., Mignogna E., Falanga A., Nicoletti G.F., Pedone C., Morelli G., Leone M., Galdiero M., and Galdiero S. 2014 Structural insights into and activity analysis of the antimicrobial peptide myxinidin. Antimicrob. Agents Chemother. 58:5280–5290. doi:10.1128/AAC.02395-14. 24957834 10.1128/AAC.02395-14 PMC4135842

[CIT0011] Chen H., Zhang M., Li B., Chen D., Dong X., Wang Y., and Gu Y. 2015 Versatile antimicrobial peptide-based zno quantum dots for in vivo bacteria diagnosis and treatment with high specificity. Biomaterials 53:532–544. doi:10.1016/j.biomaterials.2015.02.105. 25890749 10.1016/j.biomaterials.2015.02.105

[CIT0012] Chu H., Yip B., Chen K., Yu H., Chih Y., Cheng H., Chou Y., and Cheng J. 2015 Novel antimicrobial peptides with high anticancer activity and selectivity. PLoS One 10. 10.1371/journal.pone.0126390 PMC4430538 25970292

[CIT0013] Czaplewski L., Bax R., Clokie M., Dawson M., Fairhead H., Fischetti V.A., Foster S., Gilmore B.F., Hancock R.E., Harper D., et al 2016 Alternatives to antibiotics-a pipeline portfolio review. Lancet. Infect. Dis. 16:239–251. doi:10.1016/S1473-3099(15)00466-1. 26795692 10.1016/S1473-3099(15)00466-1

[CIT0014] De Smet K., and Contreras R. 2005 Human antimicrobial peptides: defensins, cathelicidins and histatins. Biotechnol. Lett. 27:1337–1347. doi:10.1007/s10529-005-0936-5. 16215847 10.1007/s10529-005-0936-5

[CIT0015] Diamond G., Zasloff M., Eck H., Brasseur M., Maloy W.L., and Bevins C.L. 1991 Tracheal antimicrobial peptide, a cysteine-rich peptide from mammalian tracheal mucosa: peptide isolation and cloning of a cdna. Proc. Natl Acad. Sci. usa 88:3952–3956. 2023943 10.1073/pnas.88.9.3952 PMC51571

[CIT0016] Dubos R.J., and Cattaneo C. 1939 studies on a bactericidal agent extracted from a soil Bacillus: III. preparation and activity of a protein-free fraction. J. Exp. Med. 70:249–256. 19870906 10.1084/jem.70.3.249 PMC2133820

[CIT0017] Environmental Agency 2005 Targeted monitoring study for veterinary medicines in the UK environment. Environment Agency, Rio House, Waterside Drive, Aztec West, Almondsbury, Bristol, BS32 4UD. https://www.gov.uk/government/uploads/system/uploads/attachment_data/file/290533/ scho0806blhh-e-e.pdf. Accessed March 13, 2014.

[CIT0018] Falla T.J., Karunaratne D.N., and Hancock R.E. 1996 Mode of action of the antimicrobial peptide indolicidin. J. Biol. Chem. 271:19298–19303. 8702613 10.1074/jbc.271.32.19298

[CIT0019] Fehlbaum P., Bulet P., Chernysh S., Briand J.P., Roussel J.P., Letellier L., Hetru C., and Hoffmann J.A. 1996 Structure-activity analysis of thanatin, a 21-residue inducible insect defense peptide with sequence homology to frog skin antimicrobial peptides. Proc. Natl Acad. Sci. usa 93:1221–1225. 8577744 10.1073/pnas.93.3.1221 PMC40060

[CIT0020] Fernandez D.I., Le Brun A.P., Whitwell T.C., Sani M.A., James M., and Separovic F. 2012 The antimicrobial peptide aurein 1.2 disrupts model membranes via the carpet mechanism. Phys. Chem. Chem. Phys. 14:15739–15751. doi:10.1039/c2cp43099a. 23093307 10.1039/c2cp43099a

[CIT0021] Frecer V., Ho B., and Ding J.L. 2004 De novo design of potent antimicrobial peptides. Antimicrob. Agents Chemother. 48:3349–3357. doi:10.1128/AAC.48.9.3349-3357.2004. 15328096 10.1128/AAC.48.9.3349-3357.2004 PMC514781

[CIT0022] de la Fuente-Núñez C., Silva O.N., Lu T.K., and Franco O.L. 2017 Antimicrobial peptides: role in human disease and potential as immunotherapies. Pharmacol. Ther. 178:132–140. 28435091 10.1016/j.pharmthera.2017.04.002

[CIT0023] Galdiero E., Siciliano A., Maselli V., Gesuele R., Guida M., Fulgione D., Galdiero S., Lombardi L., and Falanga A. 2016 An integrated study on antimicrobial activity and ecotoxicity of quantum dots and quantum dots coated with the antimicrobial peptide indolicidin. Int. J. Nanomed. 11:4199–4211. doi:10.2147/IJN.S107752. 10.2147/IJN.S107752 PMC5008656 27616887

[CIT0024] Ganz T 2002 Antimicrobial polypeptides in host defense of the respiratory tract. j. Clin. Invest. 109:693–697. doi:10.1172/JCI15218. 11901174 10.1172/JCI15218 PMC150915

[CIT0025] Gombart A.F., Borregaard N., and Koeffler H.P. 2005 Human cathelicidin antimicrobial peptide (camp) gene is a direct target of the vitamin d receptor and is strongly up-regulated in myeloid cells by 1,25-dihydroxyvitamin d3. Faseb J. 19:1067–1077. doi:10.1096/fj.04-3284com. 15985530 10.1096/fj.04-3284com

[CIT0026] Gottler L.M., and Ramamoorthy A. 2009 Structure, membrane orientation, mechanism, and function of pexiganan–a highly potent antimicrobial peptide designed from magainin. Biochim. Biophys. Acta 1788:1680–1686. doi:10.1016/j.bbamem.2008.10.009. 19010301 10.1016/j.bbamem.2008.10.009 PMC2726618

[CIT0027] Gottschalk S., Gottlieb C.T., Vestergaard M., Hansen P.R., Gram L., Ingmer H., and Thomsen L.E. 2015 Amphibian antimicrobial peptide fallaxin analogue fl9 affects virulence gene expression and dna replication in staphylococcus aureus. J. Med. Microbiol. 64:1504–1513. doi:10.1099/jmm.0.000177. 26415708 10.1099/jmm.0.000177

[CIT0028] Hajipour M.J., Fromm K.M., Ashkarran A.A., Jimenez de Aberasturi D., de Larramendi I.R., Rojo T., Serpooshan V., Parak W.J., and Mahmoudi M. 2012 Antibacterial properties of nanoparticles. Trends Biotechnol. 30:499–511. doi:10.1016/j.tibtech.2012.06.004. 22884769 10.1016/j.tibtech.2012.06.004

[CIT0029] Hara T., Kodama H., Kondo M., Wakamatsu K., Takeda A., Tachi T., and Matsuzaki K. 2001 Effects of peptide dimerization on pore formation: antiparallel disulfide-dimerized magainin 2 analogue. Biopolymers 58:437–446. doi:10.1002/1097-0282(20010405)58:4<437::AID-BIP1019>3.0.CO;2-I. 11180056 10.1002/1097-0282(20010405)58:4<437::AID-BIP1019>3.0.CO;2-I

[CIT0030] Hara T., Mitani Y., Tanaka K., Uematsu N., Takakura A., Tachi T., Kodama H., Kondo M., Mori H., Otaka A., et al 2001 Heterodimer formation between the antimicrobial peptides magainin 2 and pgla in lipid bilayers: a cross-linking study. Biochemistry 40:12395–12399. 11591159 10.1021/bi011413v

[CIT0031] Irrgang A., Roschanski N., Tenhagen B.A., Grobbel M., Skladnikiewicz-Ziemer T., Thomas K., Roesler U., and Käsbohrer A. 2016 Prevalence of mcr-1 in e. Coli from livestock and food in germany, 2010-2015. PLoS One 11:e0159863. doi:10.1371/journal.pone.0159863. 27454527 10.1371/journal.pone.0159863 PMC4959773

[CIT0032] Jensen H.H., and Hayes D.J. 2014 Impact of Denmark’s ban on antimicrobials for growth promotion. Curr. Opin. Microbiol. 19:30–36. doi:10.1016/j.mib.2014.05.020. 24997397 10.1016/j.mib.2014.05.020

[CIT0033] Jia S., Zhang X.X., Miao Y., Zhao Y., Ye L., Li B., and Zhang T. 2017 Fate of antibiotic resistance genes and their associations with bacterial community in livestock breeding wastewater and its receiving river water. Water Res. 124:259–268. doi:10.1016/j.watres.2017.07.061. 28763642 10.1016/j.watres.2017.07.061

[CIT0034] Joly S., Maze C., McCray P.B. Jr, and Guthmiller J.M. 2004 Human beta-defensins 2 and 3 demonstrate strain-selective activity against oral microorganisms. j. Clin. Microbiol. 42:1024–1029. 15004048 10.1128/JCM.42.3.1024-1029.2004 PMC356847

[CIT0035] Kazemzadeh-Narbat M., Lai B.F., Ding C., Kizhakkedathu J.N., Hancock R.E., and Wang R. 2013 Multilayered coating on titanium for controlled release of antimicrobial peptides for the prevention of implant-associated infections. Biomaterials 34:5969–5977. doi:10.1016/j.biomaterials.2013.04.036. 23680363 10.1016/j.biomaterials.2013.04.036

[CIT0036] Kolpin D.W., Furlong E.T., Meyer M.T., Thurman E.M., Zaugg S.D., Barber L.B., and Buxton H.T. 2002 Pharmaceuticals, hormones, and other organic wastewater contaminants in u.s. Streams, 1999-2000: a national reconnaissance. Environ. Sci. Technol. 36:1202–1211. 11944670 10.1021/es011055j

[CIT0037] Lai R., Zheng Y.T., Shen J.H., Liu G.J., Liu H., Lee W.H., Tang S.Z., and Zhang Y. 2002 Antimicrobial peptides from skin secretions of chinese red belly toad bombina maxima. Peptides 23:427–435. 11835991 10.1016/s0196-9781(01)00641-6

[CIT0038] Lee T.H., Hall K.N., and Aguilar M.I. 2016 Antimicrobial peptide structure and mechanism of action: a focus on the role of membrane structure. Curr. Top. Med. Chem. 16:25–39. 26139112 10.2174/1568026615666150703121700

[CIT0039] Li L.L., and Wang H. 2013 Enzyme-coated mesoporous silica nanoparticles as efficient antibacterial agents in vivo. Adv. Healthc. Mater. 2:1351–1360. doi:10.1002/adhm.201300051. 23526816 10.1002/adhm.201300051

[CIT0040] Lim S.J., Seo C.K., Kim T.H., and Myung S.W. 2013 Occurrence and ecological hazard assessment of selected veterinary medicines in livestock wastewater treatment plants. j. Environ. Sci. Health. b. 48:658–670. doi:10.1080/03601234.2013.778604. 23638893 10.1080/03601234.2013.778604

[CIT0041] Lin L., Nonejuie P., Munguia J., Hollands A., Olson J., Dam Q., Kumaraswamy M., Rivera H. Jr, Corriden R., Rohde M., et al 2015 Azithromycin synergizes with cationic antimicrobial peptides to exert bactericidal and therapeutic activity against highly multidrug-resistant gram-negative bacterial pathogens. Ebiomedicine 2:690–698. doi:10.1016/j.ebiom.2015.05.021. 26288841 10.1016/j.ebiom.2015.05.021 PMC4534682

[CIT0042] Liu Y.Y., Wang Y., Walsh T.R., Yi L.X., Zhang R., Spencer J., Doi Y., Tian G., Dong B., Huang X., et al 2016 Emergence of plasmid-mediated colistin resistance mechanism mcr-1 in animals and human beings in china: a microbiological and molecular biological study. Lancet. Infect. Dis. 16:161–168. doi:10.1016/S1473-3099(15)00424-7. 26603172 10.1016/S1473-3099(15)00424-7

[CIT0043] Marshall B.M., and Levy S.B. 2011 Food animals and antimicrobials: impacts on human health. Clin. Microbiol. Rev. 24:718–733. doi:10.1128/CMR.00002-11. 21976606 10.1128/CMR.00002-11 PMC3194830

[CIT0044] Matsunaga S., Fusetani N., and Konosu S. 1984 Bioactive marine metabolites .6. structure elucidation of discodermin-a, an antimicrobial peptide from the marine sponge discodermia-kiiensis. Tetrahedron Lett. 25:5165–5168. 10.1021/np50038a006 3839260

[CIT0045] Matsuzaki K 1998 Magainins as paradigm for the mode of action of pore forming polypeptides. Biochim. Biophys. Acta 1376:391–400. 9804997 10.1016/s0304-4157(98)00014-8

[CIT0046] Melo M.N., Ferre R., and Castanho M.A. 2009 Antimicrobial peptides: linking partition, activity and high membrane-bound concentrations. Nat. Rev. Microbiol. 7:245–250. doi:10.1038/nrmicro2095. 19219054 10.1038/nrmicro2095

[CIT0047] Näslund J., Hedman J.E., and Agestrand C. 2008 Effects of the antibiotic ciprofloxacin on the bacterial community structure and degradation of pyrene in marine sediment. Aquat. Toxicol. 90:223–227. doi:10.1016/j.aquatox.2008.09.002. 18930559 10.1016/j.aquatox.2008.09.002

[CIT0048] Nellore B.P., Kanchanapally R., Pedraza F., Sinha S.S., Pramanik A., Hamme A.T., Arslan Z., Sardar D., and Ray P.C. 2015 Bio-conjugated cnt-bridged 3d porous graphene oxide membrane for highly efficient disinfection of pathogenic bacteria and removal of toxic metals from water. acs Appl. Mater. Interfaces 7:19210–19218. doi:10.1021/acsami.5b05012. 26273843 10.1021/acsami.5b05012 PMC4690451

[CIT0049] Nordström R., and Malmsten M. 2017 Delivery systems for antimicrobial peptides. Adv. Colloid Interface Sci. 242:17–34. doi:10.1016/j.cis.2017.01.005. 28159168 10.1016/j.cis.2017.01.005

[CIT0050] Oh H., Hedberg M., Wade D., and Edlund C. 2000 Activities of synthetic hybrid peptides against anaerobic bacteria: aspects of methodology and stability. Antimicrob. Agents Chemother. 44:68–72. 10602725 10.1128/aac.44.1.68-72.2000 PMC89630

[CIT0051] O’Neil J 2014 Antimicrobial resistance: tackling a crisis for the health and wealth of nations. http://www.sfam.org.uk/download.cfm?docid=E63FDF67-7E01-4D43-8C81C619D256A18D. 2014.12. Accessed April 6, 2018.

[CIT0052] O’Neill J 2015a Rapid diagnostics stopping the unnecessary use of antibiotics. http://www.sfam.org.uk/download.cfm?docid=1191C802-F9CA-4406- 8DA9ED9E3F7B2A7F. 2015.10. Accessed April 6, 2018.

[CIT0053] O’Neill J 2015b Antimicrobials in agriculture and the environment—reducing unnecessary use and waste. http://www.sfam.org.uk/download.cfm? docid=87BCADC5-376B-42AC-A84963995CF9F5B3. 2015.12. Accessed April 6, 2018.

[CIT0054] Ottosson H., Nylén F., Sarker P., Miraglia E., Bergman P., Gudmundsson G.H., Raqib R., Agerberth B., and Strömberg R. 2016 Potent inducers of endogenous antimicrobial peptides for host directed therapy of infections. Sci. Rep. 6:36692. doi:10.1038/srep36692. 27827460 10.1038/srep36692 PMC5101518

[CIT0055] Park S., Chibli H., Wong J., and Nadeau J.L. 2011 Antimicrobial activity and cellular toxicity of nanoparticle-polymyxin b conjugates. Nanotechnology 22:185101. doi:10.1088/0957-4484/22/18/185101. 21415471 10.1088/0957-4484/22/18/185101

[CIT0056] Park S.C., Kim J.Y., Jeong C., Yoo S., Hahm K.S., and Park Y. 2011 a plausible mode of action of pseudin-2, an antimicrobial peptide from pseudis paradoxa. Biochim. Biophys. Acta 1808:171–182. doi:10.1016/j.bbamem.2010.08.023. 20826126 10.1016/j.bbamem.2010.08.023

[CIT0057] Qian H., Li J., Pan X., Sun Z., Ye C., Jin G., and Fu Z. 2012 Effects of streptomycin on growth of algae chlorella vulgaris and microcystis aeruginosa. Environ. Toxicol. 27:229–237. doi:10.1002/tox.20636. 20725941 10.1002/tox.20636

[CIT0058] Raqib R., Sarker P., Bergman P., Ara G., Lindh M., Sack D.A., Nasirul Islam K.M., Gudmundsson G.H., Andersson J., and Agerberth B. 2006 Improved outcome in shigellosis associated with butyrate induction of an endogenous peptide antibiotic. Proc. Natl Acad. Sci. usa 103:9178–9183. doi:10.1073/pnas.0602888103. 16740661 10.1073/pnas.0602888103 PMC1482586

[CIT0059] Roelants K., Fry B.G., Ye L., Stijlemans B., Brys L., Kok P., Clynen E., Schoofs L., Cornelis P., and Bossuyt F. 2013 Origin and functional diversification of an amphibian defense peptide arsenal. PLoS Genet. 9:e1003662. doi:10.1371/journal.pgen.1003662. 23935531 10.1371/journal.pgen.1003662 PMC3731216

[CIT0060] Sarmah A.K., Meyer M.T., and Boxall A.B. 2006 a global perspective on the use, sales, exposure pathways, occurrence, fate and effects of veterinary antibiotics (vas) in the environment. Chemosphere 65:725–759. doi:10.1016/j.chemosphere.2006.03.026. 16677683 10.1016/j.chemosphere.2006.03.026

[CIT0061] Schauber J., Svanholm C., Termén S., Iffland K., Menzel T., Scheppach W., Melcher R., Agerberth B., Lührs H., and Gudmundsson G.H. 2003 Expression of the cathelicidin ll-37 is modulated by short chain fatty acids in colonocytes: relevance of signalling pathways. Gut 52:735–741. 12692061 10.1136/gut.52.5.735 PMC1773650

[CIT0062] Soren O., Brinch K.S., Patel D., Liu Y., Liu A., Coates A., and Hu Y. 2015 Antimicrobial peptide novicidin synergizes with rifampin, ceftriaxone, and ceftazidime against antibiotic-resistant enterobacteriaceae in vitro. Antimicrob. Agents Chemother. 59:6233–6240. doi:10.1128/AAC.01245-15. 26248380 10.1128/AAC.01245-15 PMC4576052

[CIT0063] Steiner H., Hultmark D., Engstrom A., Bennich H., and Boman H.G. 1981 Sequence and specificity of 2 anti-bacterial proteins involved in insect immunity. Nature 292:246–248.7019715

[CIT0064] Steinmann J., Halldórsson S., Agerberth B., and Gudmundsson G.H. 2009a Phenylbutyrate induces antimicrobial peptide expression. Antimicrob. Agents Chemother. 53:5127–5133. doi:10.1128/AAC.00818-09. 19770273 10.1128/AAC.00818-09 PMC2786349

[CIT0065] Steinmann J., Halldórsson S., Agerberth B., and Gudmundsson G.H. 2009b Phenylbutyrate induces antimicrobial peptide expression. Antimicrob. Agents Chemother. 53:5127–5133. doi:10.1128/AAC.00818-09. 19770273 10.1128/AAC.00818-09 PMC2786349

[CIT0066] Tareq F.S., Lee M.A., Lee H.S., Lee Y.J., Lee J.S., Hasan C.M., Islam M.T., and Shin H.J. 2014 Gageotetrins a-c, noncytotoxic antimicrobial linear lipopeptides from a marine bacterium bacillus subtilis. Org. Lett. 16:928–931. doi:10.1021/ol403657r. 24502521 10.1021/ol403657r

[CIT0067] Taubes G 2008 The bacteria fight back. Science 321:356–361. doi:10.1126/science.321.5887.356. 18635788 10.1126/science.321.5887.356

[CIT0068] Torcato I.M., Huang Y.H., Franquelim H.G., Gaspar D., Craik D.J., Castanho M.A., and Troeira Henriques S. 2013 Design and characterization of novel antimicrobial peptides, r-bp100 and rw-bp100, with activity against gram-negative and gram-positive bacteria. Biochim. Biophys. Acta 1828:944–955. doi:10.1016/j.bbamem.2012.12.002. 23246973 10.1016/j.bbamem.2012.12.002

[CIT0069] Tossi A., Tarantino C., and Romeo D. 1997 Design of synthetic antimicrobial peptides based on sequence analogy and amphipathicity. Eur. j. Biochem. 250:549–558. 9428709 10.1111/j.1432-1033.1997.0549a.x

[CIT0070] Turner J., Cho Y., Dinh N.N., Waring A.J., and Lehrer R.I. 1998 Activities of ll-37, a cathelin-associated antimicrobial peptide of human neutrophils. Antimicrob. Agents Chemother. 42:2206–2214. 9736536 10.1128/aac.42.9.2206 PMC105778

[CIT0071] Underwood J.C., Harvey R.W., Metge D.W., Repert D.A., Baumgartner L.K., Smith R.L., Roane T.M., and Barber L.B. 2011 Effects of the antimicrobial sulfamethoxazole on groundwater bacterial enrichment. Environ. Sci. Technol. 45:3096–3101. doi:10.1021/es103605e. 21384910 10.1021/es103605e

[CIT0072] Van Boeckel T.P., Brower C., Gilbert M., Grenfell B.T., Levin S.A., Robinson T.P., Teillant A., and Laxminarayan R. 2015 Global trends in antimicrobial use in food animals. Proc. Natl Acad. Sci. USA 112:5649–5654. 25792457 10.1073/pnas.1503141112 PMC4426470

[CIT0073] Vishnuraj M.R., Kandeepan G., Rao K.H., Chand S., and Kumbhar V. 2016 Occurrence, public health hazards and detection methods of antibiotic residues in foods of animal origin: a comprehensive review. Cogent Food Agric. 2.

[CIT0074] Wade D., Boman A., Wåhlin B., Drain C.M., Andreu D., Boman H.G., and Merrifield R.B. 1990 All-d amino acid-containing channel-forming antibiotic peptides. Proc. Natl Acad. Sci. usa 87:4761–4765. 1693777 10.1073/pnas.87.12.4761 PMC54197

[CIT0075] Waghu F.H., Barai R.S., Gurung P., and Idicula-Thomas S. 2016 campr3: a database on sequences, structures and signatures of antimicrobial peptides. Nucleic Acids Res. 44(D1):D1094–D1097. doi:10.1093/nar/gkv1051. 26467475 10.1093/nar/gkv1051 PMC4702787

[CIT0076] Waghu F.H., Gopi L., Barai R.S., Ramteke P., Nizami B., and Idicula-Thomas S. 2014 camp: collection of sequences and structures of antimicrobial peptides. Nucleic Acids Res. 42:D1154–D1158. doi:10.1093/nar/gkt1157. 24265220 10.1093/nar/gkt1157 PMC3964954

[CIT0077] Wang G., Li X., and Wang Z. 2016 apd3: the antimicrobial peptide database as a tool for research and education. Nucleic Acids Res. 44(D1):D1087–D1093. doi:10.1093/nar/gkv1278. 26602694 10.1093/nar/gkv1278 PMC4702905

[CIT0078] Wang G., Mishra B., Lau K., Lushnikova T., Golla R., and Wang X. 2015 Antimicrobial peptides in 2014. Pharmaceuticals (Basel). 8:123–150. doi:10.3390/ph8010123. 25806720 10.3390/ph8010123 PMC4381204

[CIT0079] Watanabe N., Bergamaschi B.A., Loftin K.A., Meyer M.T., and Harter T. 2010 Use and environmental occurrence of antibiotics in freestall dairy farms with manured forage fields. Environ. Sci. Technol. 44:6591–6600. doi:10.1021/es100834s. 20698525 10.1021/es100834s PMC2931405

[CIT0080] Welling M.M., Brouwer C.P., van ‘t Hof W., Veerman E.C., and Amerongen A.V. 2007 Histatin-derived monomeric and dimeric synthetic peptides show strong bactericidal activity towards multidrug-resistant staphylococcus aureus in vivo. Antimicrob. Agents Chemother. 51:3416–3419. doi:10.1128/AAC.00196-07. 17620386 10.1128/AAC.00196-07 PMC2043174

[CIT0081] Yamashita T., and Saito K. 1989 Purification, primary structure, and biological activity of guinea pig neutrophil cationic peptides. Infect. Immun. 57:2405–2409. 2473036 10.1128/iai.57.8.2405-2409.1989 PMC313461

[CIT0082] Yi H., Hu W., Chen S., Lu Z., and Wang Y. 2017 Cathelicidin-wa improves intestinal epithelial barrier function and enhances host defense against enterohemorrhagic escherichia coli o157:h7 infection. J. Immunol. 198:1696–1705. doi:10.4049/jimmunol.1601221. 28062699 10.4049/jimmunol.1601221

[CIT0083] Yi H., Zhang L., Gan Z., Xiong H., Yu C., Du H., and Wang Y. 2016 High therapeutic efficacy of cathelicidin-wa against postweaning diarrhea via inhibiting inflammation and enhancing epithelial barrier in the intestine. Sci. Rep. 6:25679. doi:10.1038/srep25679. 27181680 10.1038/srep25679 PMC4867772

[CIT0084] Zasloff M 2006 Fighting infections with vitamin d. Nat. Med. 12:388–390. doi:10.1038/nm0406-388. 16598282 10.1038/nm0406-388

[CIT0085] Zhang Q.Q., Ying G.G., Pan C.G., Liu Y.S., and Zhao J.L. 2015 Comprehensive evaluation of antibiotics emission and fate in the river basins of china: source analysis, multimedia modeling, and linkage to bacterial resistance. Environ. Sci. Technol. 49:6772–6782. doi:10.1021/acs.est.5b00729. 25961663 10.1021/acs.est.5b00729

[CIT0086] Zhao X., Wu H., Lu H., Li G., and Huang Q. 2013 lamp: a database linking antimicrobial peptides. PLoS One 8:e66557. doi:10.1371/journal.pone.0066557. 23825543 10.1371/journal.pone.0066557 PMC3688957

[CIT0087] Zhu Y., Johnson T.A., Su J., Qiao M., Guo G., Stedtfeld R.D., Hashsham S.A., and Tiedje J.M. 2013 Diverse and abundant antibiotic resistance genes in Chinese swine farms. Proc. Natl Acad. Sci. USA 110:3435–3440. 23401528 10.1073/pnas.1222743110 PMC3587239

[CIT0088] Zhu X., Zhang L., Wang J., Ma Z., Xu W., Li J., and Shan A. 2015 Characterization of antimicrobial activity and mechanisms of low amphipathic peptides with different α-helical propensity. Acta Biomater. 18:155–167. doi:10.1016/j.actbio.2015.02.023. 25735802 10.1016/j.actbio.2015.02.023

